# *S*-(−)-10,11-Dihydroxyfarnesoic Acid Methyl Ester Inhibits Melanin Synthesis in Murine Melanocyte Cells

**DOI:** 10.3390/ijms150712750

**Published:** 2014-07-18

**Authors:** Seung-Hwa Baek, Jun-Won Ahn, Sung-Hee Nam, Cheol-Sik Yoon, Jae-Cheon Shin, Sang-Han Lee

**Affiliations:** 1 Department of Food Science & Biotechnology, Graduate School, Kyungpook National University, Daegu 702-701, Korea; E-Mail: micro340@hanmail.net; 2 Department of Nano-Science & Technology, Graduate School, Kyungpook National University, Daegu 702-701, Korea; E-Mail: nmrtube@naver.com; 3 Nano Convergence Practical Application Center, Daegu 704-801, Korea; 4 Department of Agricultural Biology, National Academy of Agricultural Science, Rural Administration Agency, Suwon 441-707, Korea; E-Mail: creative716@korea.kr; 5 Mycoplus Co., Ltd., Anyang 431-080, Korea; E-Mail: mycoplus@naver.com; 6 Pohang Center for Evaluation of Biomaterials, Pohang Technopark, Pohang 790-834, Korea; 7 Food & Bio-Industry Research Institute, Kyungpook National University, Daegu 702-701, Korea

**Keywords:** dihydroxyfarnesoic acid, melanin, tyrosinase, inhibitor, insect juvenile hormone, whitening

## Abstract

The development of antimelanogenic agents is important for the prevention of serious aesthetic problems such as melasmas, freckles, age spots, and chloasmas. In the course of screening for melanin synthesis inhibitors, we found that the culture broth from an insect morphopathogenic fungus, *Beauveria bassiana* CS1029, exhibits potent antimelanogenic activity. We isolated and purified an active metabolite and identified it as *S*-(−)-10,11-dihydroxyfarnesoic acid methyl ester (dhFAME), an insect juvenile hormone. To address whether dhFAME inhibits melanin synthesis, we first measured the size of the melanin biosynthesis inhibition zone caused by dhFAME. dhFAME also showed inhibitory activity against mushroom tyrosinase in Melan-a cells. Intracellular, dose-dependent tyrosinase inhibition activity was also confirmed by zymography. In addition, we showed that dhFAME strongly inhibits melanin synthesis in Melan-a cells. Furthermore, we compared levels of *TYR*, *TRP-1*, *TRP-2*, *MITF*, and *MC1R* mRNA expression by reverse-transcription polymerase chain reaction and showed that treatment of Melan-a cells with 35 μM dhFAME led to an 11-fold decrease in *TYR* expression, a 6-fold decrease in *TRP-2* expression, and a 5-fold decrease in *MITF* expression. Together, these results indicate that dhFAME is a potent inhibitor of melanin synthesis that can potentially be used for cosmetic biomaterial(s).

## 1. Introduction

Melanin pigment, which contains a combination of melanins, is derived from tyrosine, which is converted to dihydroxyphenylalanine (DOPA), then dopaquinone, and finally dopachrome by tyrosinase and other related enzyme systems [1]. Most of the current research on whitening biomaterial(s) focuses on the inhibition of melanin synthesis by targeting these enzymes [2]. Recently, *p*-methoxyphenol, hydroquinone, and kojic acid have been examined as potential whitening agents, as they inhibit melanin synthesis. However, they are not widely used as, in addition to having weak biological activity, they also have adverse effects that are lethal to skin epithelial cells [3]. Furthermore, although vitamin C and its derivatives can be used as whitening agents, the levels of tyorisnase inhibitory activity in these compounds are still not high enough for broad-spectrm use against the synthesis of the different melanins [4].

It has been shown that, in human skin and hair, there are two kinds of melanin: eumelanin and pheomelanin. Asians tend to have higher levels of eumelanin, whereas Caucasians tend to have higher levels of pheomelanin. However, as melanin pigment contains both types of melanin, the synthesis pathways for both eumelanin and pheomelanin must be inhibited. In addition, for efficient inhibition of melanin synthesis, several inhibitors targeting various steps in the synthesis pathway should be used [5]. If skin is exposed to sunlight for a long time, skin damage such as inflammation can occur; moreover, severe repeated exposure can lead to skin cancer [6]. Consequently, exposure to sunlight induces the synthesis of melanin, which protects the skin against damage from the sun. However, melanin synthesis can also lead to serious aesthetic problems such as melasmas, freckles, age spots, and chloasmas. Therefore, there is urgent demand for the development of melanin synthesis inhibitors with strong activity, particularly if they can be obtained from natural sources. Because of this, we initially performed a literature search to find potential inhibitors of melanin synthesis by looking for natural compounds that had the word “baek” (which is Korean for “white”) in their names. We isolated a metabolite from the entomopathogenic fungus *Beauveria* sp. [7], and hypothesized that it inhibits melanin synthesis [8]. This report provides evidence of the strong inhibitory activity of this metabolite against melanin synthesis via microphthalmia-associated transcription factor (MITF)-and/or tyrosinase-inhibiting machinery.

In this paper, we used column chromatography to isolate a fraction that exhibits potent antimelanogenic activity from a culture broth of the morphopathogenic insect fungus *Beauveria bassiana* CS1029. We isolated and purified the active metabolite, which we identified as *S*-(−)-10,11-dihydroxyfarnesoic acid methyl ester (dhFAME), an insect juvenile hormone. We then showed that dhFAME is a potent inhibitor of melanin biosynthesis.

## 2. Results and Discussion

### 2.1. Isolation, Purification, and Structural Determination of dhFAME

In the course of screening natural compounds for inhibitors of melanin synthesis, we found that a culture broth of *Beauveria bassiana* CS1029 exhibited potent antimelanogenic activity as determined via an *in vitro* tyrosinase inhibition assay using B16F10 cells. During the isolation and purification process, we optimized the fermentation broth culture conditions for producing the active metabolite (data not shown). We obtained 5 fractions by HP-20 column chromatography followed by silica gel chromatography and HPLC. After removing the solvent by vacuum drying, dhFAME was obtained as a freeze-dried powder. We performed NMR and HPLC for the structural determination of dhFAME.

**Figure 1:**
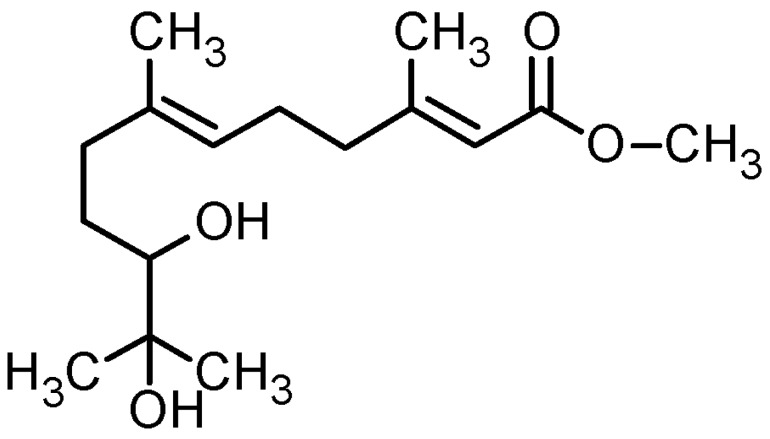
Chemical structures of *S*-(−)-10,11-dihydroxyfarnesoic acid methyl ester (dhFAME).

^1^H NMR and ^13^C NMR (500 and 125 MHz, respectively) spectra were recorded in CD_3_CN. ^1^H NMR chemical shifts are reported in parts per million relative to TMS with the solvent resonance employed as the standard (CD_3_CN at 1.98 ppm). Data are reported as follows: chemical shift, multiplicity (s = singlet, br s = broad singlet, d = doublet, br d = broad doublet, t = triplet, br t = broad triplet, q = quartet, m = multiplet), coupling constants (Hz) and integration. ^13^C NMR chemical shifts are reported in ppm from TMS with the solvent resonance employed as the standard CD_3_CN at 0.5 ppm. The structure determination of dhFAME was performed by HPLC analysis using a Shim-packv VP-ODS (4.6 × 250 mm, particle size 5 μm, Shimadzu, Kyoto, Japan) column (100% acetonitrile; flow rate; 1 mL/min; λ = 254 nm; *t*_EF-1_ = 7.66 min); ^1^H NMR (500 MHz, CD_3_CN), δ (ppm): 1.09 (s), 1.11 (s), 1.30 (m), 1.61 (m), 1.64 (br s), 2.02 (m), 2.17 (br d), 2.24 (m), 2.25 (m), 2.66 (br s), 2.82 (br d), 3.20 (br), 3.65 (s), 5.17 (br t), and 5.70 (br s); ^13^C NMR (125 MHz, CD_3_CN), δ (ppm): 15.3, 18.0, 23.7, 25.2, 25.7, 29.9, 36.6, 40.4, 50.4, 72.3, 77.6, 115.2, 123.3, 136.3, 160.3, and 167.0 (Table 1). NMR analysis was carried out by injecting CDCl_3_-dissolved samples in 5 mm tubes. Careful interpretation of the mass spectrometry and NMR spectroscopy data revealed that the substance was *S*-(−)-10,11-dihydroxyfarnesoic acid methyl ester (dhFAME, Figure 1).

**Table 1:** Structure determination of dhFAME.

Position	δ_H_ (ppm ^a^) Multi ( *J* in Hz)		δ_C_ (ppm ^a^)	
1			167.0	C
2	5.70	br s	115.2	CH
3			160.3	C
4	2.24	m	40.4	CH_2_
5	2.24	m	25.7	CH_2_
6	5.17	br t (6.5)	123.3	CH
7			136.3	C
8	2.25	m	36.6	CH_2_
	2.02	m		
9	1.61	m	29.9	CH_2_
	1.30	m		
10	3.20	br ddd	77.6	CH_2_
11			72.3	C
12 ^b^	1.11	s	25.2	CH_3_
13 ^b^	1.09	s	23.7	CH_3_
14	1.64	br s	15.3	CH_3_
15	2.17	br d (1.0)	18.0	CH_3_
OCH_3_	3.65	s	50.4	CH_3_
10-OH	2.82	br d (5.0)		
11-OH	2.66	br s		

^a^ ^1^H and ^13^C chemical shifts in ppm from solvent references (CD_3_CN δ_H_ 1.98, δ_C_ 0.5); ^b^ Assignments of C-12 and C-13 could be interchanged.

It is known that hyperpigmentation as well as skin inflammation can be caused by hormonal abnormalities, genetic disorders, and ultraviolet irradiation [9]. Although the presence of melanin in the skin protects dermal cells from ultraviolet light [10], the overproduction of melanin can cause liver spots or freckles to form. Therefore, a surplus of melanin will cause skin to appear older and ultimately lead to skin cancer. In recent years, the development of cosmetics/cosmeceuticals to prevent melanin overproduction has been accelerated [11]. Insect juvenile hormone is composed of aliphatic terpenes [12]. It has epoxide hydrolase activity, which functions in the biosynthesis of the hormone, and is known to play a key role in metamorphosis [13]. In the course of screening for antimelanogenic agents, we interestingly found dhFAME, an insect juvenile hormone that is produced by *Beauveria bassiana* CS1029. We have little information as to what role the activation of juvenile hormone epoxide hydrolase may play in *Beauveria bassiana* CS1029. Moreover, we have no data indicating why the fungus excretes this substance into the medium, as this study focuses on the inhibition of melanin synthesis caused by *Beauveria* sp*.*

### 2.2. Effects of S-(−)-10,11-Dihydroxyfarnesoic Acid Methyl Ester (dhFAME) on Melanin Biosynthesis in Streptomyces Bikiniensis and on Tyrosinase Activity

**Figure 2:**
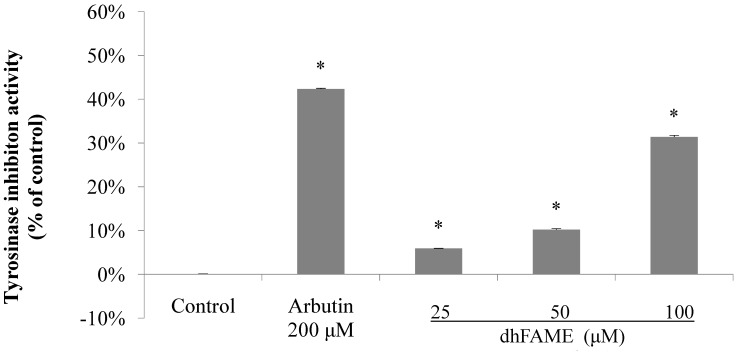
Effect of dhFAME against mushroom tyrosinase. Tyrosinase was preincubated with test substances at 25 °C for 5 min prior to incubation with l-tyrosine for 30 min, and absorbance was read at 490 nm. Each determination was made in triplicate, and the data shown represent the mean ± standard deviation. Statistical significance (* *p* < 0.05) was determined using Student’s *t-*test.

Next, we analyzed the inhibitory effects of dhFAME on melanogenesis by *Streptomyces bikiniensis* using the paper-disc diffusion method. The inhibition zone surrounding each paper disc showed clear inhibitory activity between 25 and 100 μg/mL in *Streptomyces bikiniensis* (data not shown). The results showed that dhFAME potently inhibited melanin biosynthesis in a concentration-dependent manner (data not shown). An *in vitro* tyrosinase assay also showed that the metabolite had potent inhibitory activity. As shown in Figure 2, dhFAME clearly inhibited tyrosinase activity in a concentration-dependent manner: dhFAME reduced the levels of activity to 5.6%, 10.0%, and 30.8% that of the control at 25, 50, and 100 μM, respectively, whereas arbutin only reduced the level of activity to 42.2% that of the control at 200 μM. Arbutin has been reported to inhibit melanin biosynthesis at a concentration of 500 μM. However, the level of inhibition exhibited by dhFAME was 1.5 times higher than that of arbutin, as shown in Figure 2. Our results indicate that, even at low concentrations, the present metabolite is a promising whitening agent.

It is well known that fermentation broths produced by *Beauveria bassiana* CS1029 have the potential to yield powerful cosmetic biomaterials because this strain produces several natural compounds. Nevertheless, the toxicity of many fungal metabolites is problematic. One such metabolite is kojic acid, a pyrone derivative, which is obtained from the fermentation of Japanese liquor. Although a formulation containing 1% kojic acid was shown to be effective in preventing hyperpigmentation, the use of this compound for skin whitening has come to a standstill because of concerns about its potential carcinogenic effects [14,15].

### 2.3. Effect of dhFAME on Cell Viability and Melanin Content

**Figure 3:**
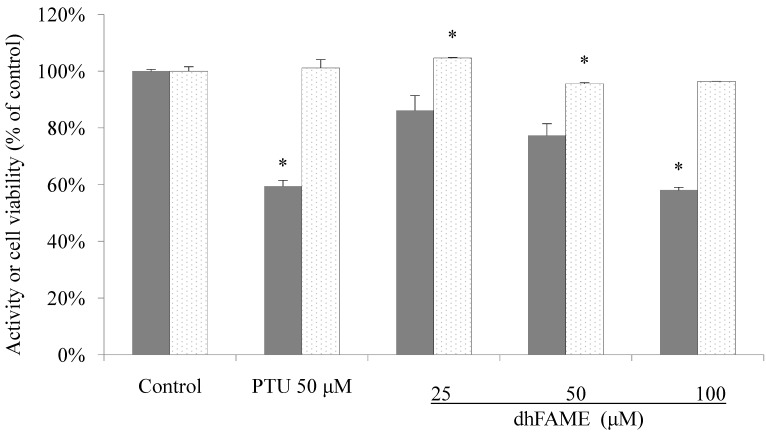
Effect of dhFAME on melanogenesis and cell viability in Melan-a cells. Cells (1 × 10^5^ cells/mL) were cultured for 24 h, and the medium was replaced with fresh medium containing various concentrations of compound and PTU (phenylthiourea) for 3 days. The cells were collected and lysed with 1 N NaOH. The melanin contents were estimated by measuring the absorbance at 405 nm. Each determination was made in triplicate, and the data shown represent the mean ± standard deviation. Statistical significance (* *p* < 0.05) was determined using Student’s *t-*test.

We directly measured *in vitro* melanin content and cell viability in Melan-a cells after dhFAME treatment. The results showed that cells treated with 100 μM dhFAME did not exhibit either cytotoxicity or morphological changes as compared to control cells, although the melanin content in the cells was significantly decreased to 41.6% that of the control (Figure 3, 1st–5th white columns). We performed the standard *in vivo* toxicity tests, including phototoxicity, skin irritation toxicity, and eye irritation assay, and showed that dhFAME exhibited no toxicity at a designated concentration of 100 mg/dose [16] and data not shown.

Toxicity can be evaluated via three tests: (1) the eye mucosa irritation test; (2) the skin irritation test; and (3) the phototoxicity test. These tests are commonly used standard toxicity tests recommended by the Korea Food and Drug Administration, and all cosmetic products must be cleared via these tests before market release, although alternative tests have also been used recently. We evaluated the biosafety of dhFAME using these tests to determine if it could be used for cosmetic purposes. The results revealed that dhFAME did not exhibit any toxicity in rabbit or guinea pig animal models (data not shown). In addition, we examined cell toxicity using Melan-a cells. The data indicated that dhFAME did not cause any remarkable damage on the cellular level (Figure 3, white columns). After confirming the biosafety of dhFAME using the above tests, we then investigated its effect on signaling pathways involved in melanogenesis.

### 2.4. Effects of dhFAME on Zymography, Tyrosinase Assay and the Expression of Melanogenesis-Related Genes

**Figure 4:**
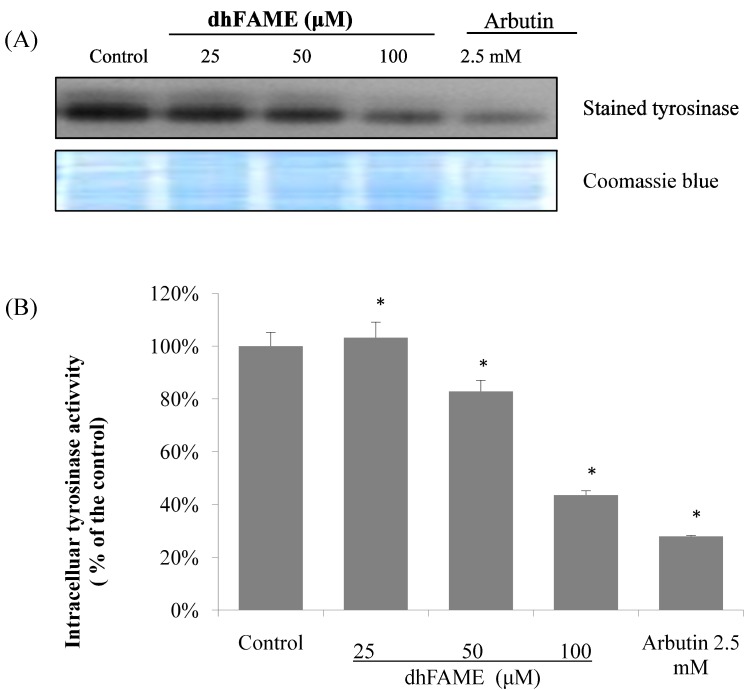
Effect of dhFAME on intracellular tyrosinase activity in Melan-a cells. (**A**) l-3,4-dihydroxyphenylalanine (l-DOPA) zymography analysis of the inhibition of Melan-a tyrosinase by dhFAME. Cells (1 × 10^5^ cells/mL) were cultured for 24 h, and then the medium was replaced with fresh medium containing various concentrations of compound and arbutin for 3 days. The cells were then collected and lysed. After the protein levels were quantified, tyrosinase activity was determined by l-DOPA zymography. The proteins were visualized by Coomassie blue staining; (**B**) Cells were cultured under the same conditions as above. The cell lysates were incubated with 20 mM l-DOPA for 1 h. Each determination was made in triplicate, and the data shown represent the mean ± standard deviation. Statistical significance (* *p* < 0.05) was determined using Student’s *t-*test.

**Figure 5:**
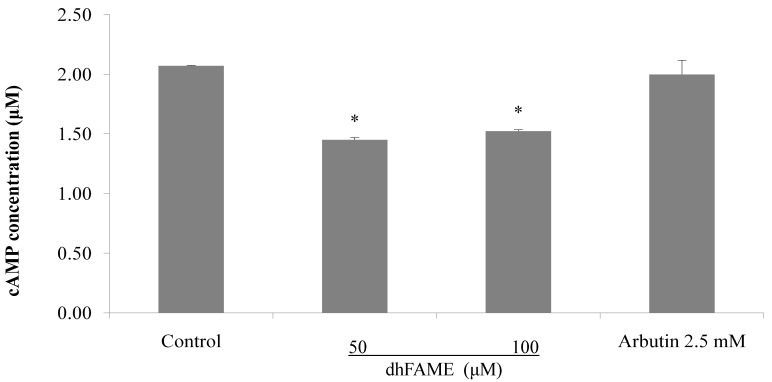
Effect of dhFAME on intracellular cAMP concentration in Melan-a cells. Cells (3 × 10^5^ cells/well) were cultured for 24 h and the medium was replaced with fresh medium containing various concentrations of dhFAME and arbutin for 12 h. Intracellular cAMP levels were measured using a cAMP direct immunoassay kit. Each determination was made in triplicate and the data shown represent the mean ± SD. Data was considered to indicate statistical significance (* *p* < 0.05) by means of the student *t*-test.

The effects of dhFAME on tyrosinase as measured by l-3,4-dihydroxyphenylalanine (L-DOPA) zymography [17] are shown in Figure 4. Treatment with dhFAME at concentrations ranging from 25 to 100 µM resulted in increasing degrees of inhibition of tyrosinase in Melan-a cells (Figure 4a; compare 1st to 2nd–4th bands). When cellular tyrosinase inhibitory activity was measured in Melan-a cells, dhFAME showed a potent antityrosinase effect with an IC_50_ = 97 µM (Figure 4b), which confirmed the results from the l-DOPA zymography. In addition, dhFAME inhibited the intracellular cyclic AMP (cAMP) levels in Melan-a cells (Figure 5).

**Figure 6:**
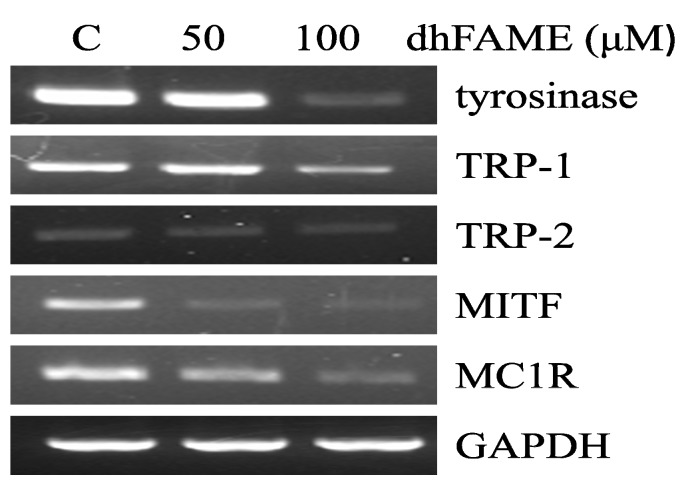
Effect of dhFAME on mRNA expression of melanogenesis-related genes in Melan-a cells. The cells were cultured for 24 h, and then the medium was replaced with fresh medium containing various concentrations (50 and 100 μM) of methyl ester for 24 h. The mRNA levels were then analyzed by reverse transcriptase polymerase chain reaction. The band intensity was normalized to GAPDH rRNA using an Image J program [18].

Next, to investigate which melanogenesis-related genes were affected by dhFAME, we used reverse transcriptase-PCR to measure the levels of *TYR*, *TRP-1*, *TRP-2*, *MITF*, and *MC1R* mRNA in Melan-a cells. dhFAME suppressed the expression of *TYR*, *TRP-1*, and *TRP-2*, with 90%, 50%, and 30% inhibition observed at 100 μM dhFAME, respectively (Figure 6). Additionally, dhFAME significantly decreased the expression of *MC1R*, which encodes a receptor on the surface of melanocytes, and MITF. These results clearly indicate that dhFAME may contribute to the inhibition of melanogenesis by regulating the expression of tyrosine-related genes as well as *MITF* and *MC1R*.

Melanin pigmentation in human skin has complex regulation with different stages [19]. Melanogenesis-related signaling genes include *TYR*, *TRP-1*, *TRP-2*, *MITF*, *MC1R*, *ASIP*, and others. The red hair and fair skin phenotype in humans frequently arises from genetic variants in *MC1R* [20]. In mice, MC1R in hair follicle melanocytes regulates coat color. Activation of MC1R by the ligand α-mealnocyte stimulating hormone (α-MSH) results in the production of the black pigment eumelanin, whereas suppression of MC1R by the inverse agonist Agouti promotes the production of the yellow pigment pheomelanin. MGRN1 may ubiquitinate either MC1R or a downstream target, complementing Agouti signaling in non-mutant mice. Reduction or loss of MGRN1-mediated ubiquitination in Mgrn1md-nc mutant mice stimulates the production of eumelanin, producing a darker coat [21]. The melanogenic proteins, such as tyrosinase, TRP-1 and -2, are regulated by a specific transcription factor, microphthalmia-associated transcription factor (MITF). MITF possesses a basic helix–loop–helix structure for DNA binding, and plays a fundamental role in the transcriptional regulation of melanogenesis [22]. Endogenous cyclic AMP elevating stimuli such as α-MSH and ACTH promotes the expression of MITF through the activation of protein kinase A (PKA) and phosphorylates cAMP-related binding protein (CREB), which binds to the CRE consensus motif in the promoter region of MITF [23,24]. Our results show that dhFAME treatment dramatically reduced the expression of *MITF* mRNA as well as *MC1R* mRNA. Moreover, dhFAME (100 µM) reduced the intracellular cAMP levels by comparison with control. These results clearly demonstrated that dhFAME decreases melanogensis via the cAMP pathway through the down-regulation of *MC1R* gene expression, which subsequently decreases the *MITF*, *TYR*, *TRP-1*, *TRP-2* gene expression.

In summary, in our present study, we show that dhFAME, a whitening agent purified from an insect morphopathogenic fungus, has no toxicity in three recommended toxicity tests. The compound is thus promising for the development of skin whitening ingredient(s) and/or biomaterial(s) in the cosmetic/cosmeceutical industry.

## 3. Experimental Section

### 3.1. Materials

Arbutin, αmelanin stimulating hormone (MSH), and IBMX (3-isobutyl-1-methylxanthine; 1-methyl-3-(2-methylpropyl)-7*H*-purine-2,6-dione) were obtained from Sigma Co. (St. Louis, MO, USA). The melanocyte line, Melan-a, was obtained from D.C Bennett (St George’s University of London, London, UK). All other reagents were commercially available.

### 3.2. Purification and Structure Determination of S-(−)-10,11-Dihyroxyfarnesic Acid Methyl Ester (dhFAME)

Sabourand Dextrose Agar (BBL, 500 g/bt; Franklin Lakes, NJ, USA) was used to grow *Beauveria bassiana* CS1029 cells in a slant at 27 °C for 1 week, and cultured in a sterile liquid medium (Difco, 238230; Franklin Lakes, NJ, USA) by inoculating cell clumps of CS1029 (8 × 8 mm) in a shaking incubator (27 °C, 180 rpm) for 4 day [19]. After centrifugation (2000 rpm, 10 min) of fermentation broth (total volume of 1000 mL), supernatants were collected and applied to HP-20 column (100 × 1300 mm) using a developing solvent system. After washing with 35% ethanol (3 volumes of broth), the active compound that inhibits melanin synthesis was extracted with 80% ethanol four times. Concentrations on silica gel (Silicagel, 55 × 800 mm, 0.40~0.63 μm) using 2nd extractions were conducted. Chloroform and ethyl acetate were used at 100:10, 100:20, and 100:30 to develop the production of the active fractions collected. Fractions were collected by a high-performance liquid chromatograph (HPLC, Shimadzu LC-6AD, Kyoto, Japan). Analysis conditions for Shim-pack Prep-ODS (H) Kit 250 × 20 mm column used were ultrapure water in acetonitrile. During the procedure, the dhFAME (of 5 fractions) was isolated with a flow rate of 5 mL/min at room temperature using PDA detector (Shimadzu SPD-M10Avp, Kyoto, Japan). After removing the solvent by vacuum drying, dhFAME was obtained as freeze-dried powder. NMR analysis (Varian Inova-300, Crawley, Australia) was carried out by injection of CDCl_3_-dissolved sample to put in 5 mm tubes, using TMS as internal standard reagent grade. Careful interpretation of the data of mass spectrometry and NMR spectroscopy revealed that the whitening substance was *S*-(−)-10,11-dihyroxyfarnesic acid methyl ester (dhFAME) [7].

### 3.3. Measurement of Tyrosinase Activity

The activities of tyrosinase were spectrophotometrically determined as described previously with minor modification [21]. The reaction mixture for the determination of mushroom tyrosinase (EC:1.14.18.1) activity contained 150 μL of 0.1 M phosphate buffer (pH 6.5), 3 μL of sample, 36 μL of 1.5 mM l-tyrosine and 7 μL of mushroom tyrosinase (2100 units/mL, 0.05 M phosphate buffer, pH 6.5) in a 96-well microplate (SPL, Pocheon, Korea). The mixture was measured at 490 nm for initial value. After incubation at 37 °C for 30 min, absorbance was measured at 490 nm using a microplate reader (VICTOR3, Perkin Elmer, Waltham, MA, USA).

Inhibition activity (%) = ((*A* − *B*) − (*C* − *D*))/(*A* − *B*) × 100 (1)

where *A*: Absorbance of control treatment after reaction; *B*: Absorbance of control treatment before reaction; *C*: Absorbance of sample treatment after reaction; and *D*: Absorbance of sample treatment before reaction.

### 3.4. Cell Culture

The Melan-a cells were cultured in RPMI 1640 supplemented with 10% fetal bovine serum (FBS; HyClone, Logan, UT, USA), streptomycin-penicillin (100 μg/mL each), and 200 nM tetradeconyl phorbol acetate (TPA), a potent tumor promoter, at 37 °C in 5% CO_2_. Cells were passed every 3 days until a maximal passage number of 40. Confluent monolayers of melanocytes were harvested with a mixture of 0.05% trypsin and 0.53 mM EDTA (Gibco BRL, Grand Island, NY, USA).

### 3.5. Cell Viability Assay

Cell viability after incubation for an additional 3 days was determined using a MTT assay [25]. Various concentrations of sample were added to the cells. Next, 100 μL of MTT solution (5 mg/mL MTT in PBS) was added to each well, followed by incubation at 37 °C for 1 h. After removal of MTT solution, 1000 μL of dimethyl sulfoxide was added into wells with vigorous mixing. Absorbance was determined with a microplate reader (VICTOR3, Perkin Elmer, Waltham, MA, USA) at 470 nm.

### 3.6. Melanization Inhibition Assay on Melan-a Cells

Cells were seeded into a 24-well plate (BD Falcon, Bedford, MA, USA) at a density of 1 × 10^5^ cells per well, and allowed to attach overnight. The medium was replaced with fresh medium containing various concentrations of compound for 72 h. After washing them with phosphate-buffered saline (PBS), the cells were lysed with 250 μL of 1 N NaOH and transferred to a 96-well plate. The melanin contents were estimated by measuring the absorbance at 405 nm using a microplate counter (VICTOR3, Perkin Elmer, Waltham, MA, USA) as described elsewhere [26]. Phenyltuiourea (PTU) was used as a positive control. The results were calculated as the amount of intracellular melanin per cell and expressed as percentage of control.

### 3.7. Analysis of Tyrosinase Activity by Zymography

Tyrosinase zymography was performed as described elsewhere [27,28]. Test compounds were added to Melan-a cells (1 × 10^5^ cells/well; 24-well plates), and the culture cells were washed with PBS and harvested with RIPA cell lysis buffer supplemented protease inhibitor. The amount of protein contents was determined with a BCA protein assay kit (Sigma, St. Louis, MO, USA). Equal amounts (40 μg protein) of each sample were mixed with zymogram sample buffer at 37 °C for 30 min, and samples were loaded on a 10% SDS-PAGE gel (sodium dodecyl sulfate-polyacrylamide gel electrophoresis). After electrophoresis, the gel was incubated with 0.1 M sodium phosphate buffer for 30 min by gentle shaking. The gel was stained to 20 mM l-DOPA in 0.1 M sodium phosphate buffer at 37 °C for 1 h.

### 3.8. Reverse-Transcription Polymerase Chain Reaction (RT-PCR) Analysis of mRNA Expression

Total RNA was extracted using a TRI-zol (Invitrogen, Carlsbad, CA, USA) according to the manufacturer’s instructions [29]. The quality of the total RNA sample was evaluated by determining the OD_260_/OD_280_ ratio. To prepare a cDNA pool from each RNA sample, total RNA (2 μg) was reverse transcribed at 42 °C for 90 min in the presence of oligo (dT) primers and reverse transcriptase (Roche Molecular Biochemicals, Mannheim, Germany). The initial denaturation step was performed for 2 min at 95 °C and amplified at 30 s at 94 °C, 30 s at 56 °C, and 45 s at 72 °C for 29 cycles, followed by a 5 min elongation cycle at 72 °C using a PCR Thermal Cycler Dice TP600 (TAKARA Bio Inc., Otsu, Japan). The oligonucleotides primers for mouse tyrosinase (forward, 5'-CCCAGAAGCCAATGCACCTA-3'; reverse, 5'-ATAACAGCTCCCACCAGTGC-3'), mouse TRP-1 forward, 5'-GCTGCAGGAGCCTTCTTTCT-3'; reverse, 5'-AAGACGCTGCACTGCTGGTC-3'), mouse TRP-2 forward, 5'-GGATGACCGTGAGCAATGGC-3'; reverse, 5'-CGGTTGTGACCAATGGGTGC-3'), mouse MITF forward, 5'-CAGGCTAGAGCGCATGGACT-3'; reverse, 5'-CTCCGTTTCTTCTGCGCTCA-3'), mouse MC1R forward, 5'-ATCCCAGATGGCCTCTTCCT-3'; reverse, 5'-ACACCATGGAGCCACAGATG-3') and mouse 18S rRNA as an internal control (forward, 5'-AGAAACGGCTACCACATCCA-3'; reverse, 5'-TACGCTATTGGGGCTGGAAT-3') were used. The PCR products were electrophoresed at 100 V for 40 min on a 2% agarose gel in TBE buffer. After electrophoresis, PCR products were visualized by ethidium bromide staining and the signal intensity of each band was quantified and normalized GAPDH. The data were quantified with Image J software [18].

### 3.9. Measurement of cAMP

Cellular cAMP assay was performed with cell lysate using a cAMP direct immunoassay kit (Biovision, San Francisco, CA, USA). Cells were split into 6-well plates at 3 × 10^5^ cells/well and incubated at 37 °C in normal culture conditions overnight [30]. Cells were treated with the compound at various concentrations, and the cells were lysed and processed according to the manufacturer’s protocol using 0.1 N HCl. cAMP measurements were carried out as described in the cAMP kit manual using a microplate counter (VICTOR3, Perkin Elmer, Waltham, MA, USA).

### 3.10. Statistical Analysis

Data were expressed as the means ± standard deviation of the mean values. Statistical significance was determined by a Student’s *t*-test for independent means, using the Microsoft Excel program. The critical level for significance was set at *p* < 0.05.

## 4. Conclusions

We identified an active metabolite as *S*-(−)-10,11-dihydroxyfarnesoic acid methyl ester (dhFAME) from the culture broth from an insect morphopathogenic fungus, *Beauveria bassiana* CS1029. dhFAME strongly inhibits not only tyrosinase activity, but also melanin synthesis without cytotoxicity in Melan-a cells. Furthermore, treatment with 35 μM dhFAME led to an 11-fold decrease in *TYR* expression, a 6-fold decrease in *TRP-2* expression, and a 5-fold decrease in *MITF* expression. Levels of cAMP in Melan-a cells were decreased by dhFAME treatement in comparison with control. These data suggest that dhFAME may inhibit melanogensis through a MC1R/cAMP/MITF signaling pathway. Overall, it appears that dhFAME is a potent inhibitor of melanin synthesis that can potentially be used for cosmetic biomaterial(s).
